# Pyrotinib and chrysin synergistically potentiate autophagy in HER2-positive breast cancer

**DOI:** 10.1038/s41392-023-01689-w

**Published:** 2023-12-18

**Authors:** Xiaoxiao Liu, Xing Zhang, Zhiying Shao, Xiaorong Zhong, Xin Ding, Liang Wu, Jie Chen, Ping He, Yan Cheng, Kunrui Zhu, Dan Zheng, Jing Jing, Ting Luo

**Affiliations:** 1 Institute for Breast Health Medicine, Cancer Center, Breast Center, West China Hospital, Sichuan University, 610041 Chengdu, Sichuan China; 2 Department of Radiation Oncology, Cancer Center, Affiliated Hospital of Xuzhou Medical University; Jiangsu Center for the Collaboration and Innovation of Cancer Biotherapy, Cancer Institute, Xuzhou Medical University, 221000 Xuzhou, China; 3 Department of Orthopedics, Trauma and Reconstructive Surgery, University Hospital RWTH Aachen, Aachen, 52074 Germany; 4 Cancer Institute, Xuzhou Medical University, 221000 Xuzhou, Jiangsu China; 5 Division of Nephrology and Transplantation, Department of Internal Medicine, University Medical Center Rotterdam Erasmus MC, Rotterdam, 3015 GD The Netherlands; 6 Institute for Breast Health Medicine, Department of General Surgery, Breast Center, West China Hospital, Sichuan University, 610041 Chengdu, Sichuan China; 7 Institute for Breast Health Medicine, West China Hospital, Sichuan University, 610041 Chengdu, Sichuan China

**Keywords:** Breast cancer, Breast cancer

## Abstract

Human epidermal growth factor receptor 2 (HER2)-positive breast cancer (BC) has been the most challenging subtype of BC, consisting of 20% of BC with an apparent correlation with poor prognosis. Despite that pyrotinib, a new HER2 inhibitor, has led to dramatic improvements in prognosis, the efficacy of pyrotinib monotherapy remains largely restricted due to its acquired resistance. Therefore, identifying a new potential antitumor drug in combination with pyrotinib to amplify therapeutic efficacy is a pressing necessity. Here, we reported a novel combination of pyrotinib with chrysin and explored its antitumor efficacy and the underlying mechanism in HER2-positive BC. We determined that pyrotinib combined with chrysin yielded a potent synergistic effect to induce more evident cell cycle arrest, inhibit the proliferation of BT-474 and SK-BR-3 BC cells, and repress in vivo tumor growth in xenograft mice models. This may be attributed to enhanced autophagy induced by endoplasmic reticulum stress. Furthermore, the combined treatment of pyrotinib and chrysin induced ubiquitination and glucose-6-phosphate dehydrogenase (G6PD) degradation by upregulating zinc finger and BTB/POZ domain-containing family protein 16 (ZBTB16) in tumorigenesis of BC. Mechanistically, we identified that miR-16-5p was a potential upstream regulator of ZBTB16, and it showed a significant inverse correlation with ZBTB16. Inhibition of miR-16-5p overexpression by restoring ZBTB16 significantly potentiated the overall antitumor efficacy of pyrotinib combined with chrysin against HER2-positive BC. Together, these findings demonstrate that the combined treatment of pyrotinib and chrysin enhances autophagy in HER2-positive BC through an unrecognized miR-16-5p/ZBTB16/G6PD axis.

## Introduction

Breast cancer is one of the most prevalent types of malignant tumors that threaten worldwide women’s health. Recent data have shown that breast cancer has become the leading cause of death among women aged 20–50.^1^ HER2, a member of the epidermal growth factor receptor family, has tyrosine kinase activity, and the amplification of HER2 occurring in breast cancer is defined as HER2-positive breast cancer.^2^ Although HER2-positive breast cancer accounts for only around 20% of all breast cancer cases, it remains the most intractable subtype of breast cancer in clinical practice.^3^ HER2-positive breast cancers are characterized by high invasive capability and metastatic potential, which is associated with poor prognosis of patients.^4^ Despite that a variety of targeted drugs have been developed for HER2-positive breast cancer, anti-HER2 resistance has evolved as a primary cause of therapeutic failure in HER2-positive breast cancer patients.^3,5^ Therefore, there is an urgent demand to explore drug resistance mechanisms and identify new combinations of targeted drugs as an alternative regimen that synergistically reinforces the antitumor efficacy.

Pyrotinib, an oral irreversible dual-ErbB tyrosine kinase inhibitor (TKI) approved by the Chinese State Drug Administration, was developed by Shanghai Hengrui Medicine for treating HER2 hyperactive breast cancer.^6^ At present, pyrotinib has been proven to be clinically effective in solid tumors including breast cancer, gastric cancer, and non-small cell lung cancer.^7–9^ A number of preclinical and clinical studies have demonstrated the utility of pyrotinib in the treatment of HER2-positive cancers.^5,10^ A phase II randomized trial reported that women with HER2-positive metastatic breast cancer treated with pyrotinib plus capecitabine yielded significantly better objective response rates and survival outcomes than lapatinib plus capecitabine.^11^ Consistent with this, the analysis based on individual patient-level data from three clinical trials revealed that the combination treatment of pyrotinib plus capecitabine could dramatically improve the objective response rate and progress-free survival in HER2-positive metastatic breast cancer patients.^12^ However, the clinical effectiveness of pyrotinib monotherapy is somewhat restricted due to its drug resistance, and it is expected that the future trend in treating HER2-positive breast cancer will be toward the combination of pyrotinib with another potent antitumor drug, which is urgently needed for potentiating therapeutic efficacy in clinical practice.

Chrysin, also known as 5, 7-dihydroxyflavone, is a flavonoid extracted from the Bignoniaceae plant oryx and is relatively high in propolis.^13,14^ It has a wide range of pharmacological and physiological activities such as anti-oxidation, antitumor, anti-hypertension, anti-diabetes, anti-bacteria, and anti-allergy.^15,16^ In view of its wide distribution and relatively low toxicity, chrysin has been considered a promising therapeutic agent for various diseases.^13,17,18^ Recently, a series of studies have shown that chrysin has anti-proliferative and pro-apoptotic activities in various cancers including leukemia, and cervical, esophageal, prostate, thyroid and breast cancers,^19,20^ manifesting its great potential as an antitumor agent. Importantly, our previous studies have uncovered its role in hepatocellular carcinoma by regulating endoplasmic reticulum (ER) stress, thereby influencing tumor progression.^21^ From the therapeutic point of view, it is surprising that the majority of recent studies have failed to note the enormous value of chrysin on HER2-positive breast cancer.^22,23^ Moreover, no data so far is available regarding the efficacy of pyrotinib combined with chrysin in treating HER2-positive breast cancer. Therefore, we speculate that pyrotinib in combination with chrysin could exert a potent synergistic antitumor effect against HER2-positive breast cancer.

In this study, we investigated the anticancer efficacy of pyrotinib in combination with chrysin as a novel therapeutic regimen and explored the underlying molecular mechanisms in the treatment of HER2-positive breast cancer. The results suggest that the combined treatment of pyrotinib and chrysin synergistically inhibits HER2-positive breast cancer cell survival and proliferation in vitro and in vivo by augmenting autophagy, which is accomplished through the miR-16-5p/ZBTB16/G6PD axis. Together, this work provides novel insights into developing a potent combination therapy against HER2-positive breast cancer.

## Results

### Combination of chrysin plus pyrotinib yields a synergistic therapeutic superiority to inhibit HER2-positive breast cancer in vitro and in vitro

**Fig. 1:**
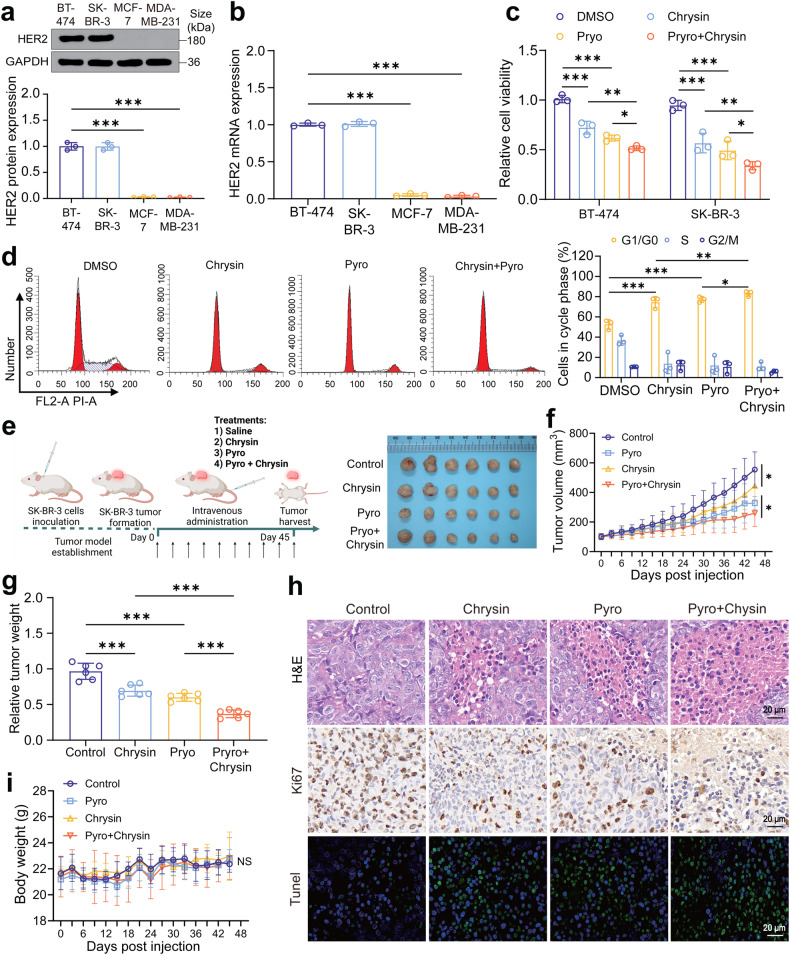
Therapeutic effect of pyrotinib and chrysin on HER2-positive breast cancer in vitro and in vivo. **a**, **b** Western blot (**a**) and RT-qPCR (**b**) analysis of HER2 expression levels in various breast cancer cell lines of MCF-7, SK-BR-3, BT-474, MDA-MB-231 (*n* = 3). **c** Cell viability of SK-BR-3 cells received various treatments (*n* = 3). **d** Detection of cell cycle arrest of SK-BR-3 cells after various treatments (*n* = 3). **e** Schematic diagram of the established protocol of the animal models. Photograph of resected tumor tissues of mice in different treatment groups during the whole testing period (*n* = 6). **f**, **g** Tumor volume (**f**) and tumor weight (**g**) variations of mice in four treatment groups over 45 days (*n* = 6). **h** H&E, Ki67, and Tunnel immunohistochemical staining of tumor sections of mice in various treatment groups. **i** body weight changes in different treatment groups during the whole testing period. All bar values are represented as mean ± SD. *P < 0.05, **P < 0.01, and ***P < 0.001. NS, not significant

First, we examined the therapeutic efficacy of pyrotinib plus chrysin against HER2-positive breast cancer in vitro and in vivo. Western blot and RT-qPCR results showed that high expression of HER2 occurred in both BT-474 and SK-BR-3 cell lines, and these two were therefore selected for the subsequent experiments (Fig. 1a, b). To examine the antitumor efficacy of the combination therapy of pyrotinib plus chrysin against HER2-positive breast cancer, BT-474 and SK-BR-3 cells were treated with chrysin-only, pyrotinib-only or combination treatment of chrysin with pyrotinib. It was noteworthy that three treatment groups produced varying degrees of antitumor effect in comparison to the control group (DMSO), and the combined therapy group elicited the most inhibitory efficacy in cell viability (Fig. 1c). Meanwhile, the proportion of SK-BR-3 cells arrested in the G1/G0 phase pointedly increased after treatment with either chrysin or pyrotinib alone, but the blocking effect was the most significant in the combination group (Fig. 1d). Motivated by in vitro gratifying therapeutic efficacy of pyrotinib plus chrysin, SK-BR-3 tumor xenograft mouse models were established to investigate in vivo antitumor efficacy of the two drugs on tumor growth either individually or synergistically. The results showed that solely chrysin or pyrotinib treatment demonstrated a limited degree of the inhibition of tumor volume and tumor mass, while the combined group showed the highest inhibitory effect among all treatment groups (Fig. 1e–g). To get a deep insight into the superiority of the combination treatment, H&E and Ki67 immunohistochemical staining were conducted. Notably, significantly decreased levels of Ki67 were observed in all treatment groups (Fig. 1h), suggesting varying degrees of inhibitory effect on tumor cell proliferation. Tunnel staining also revealed a significantly higher apoptosis level in the combination group than that in chrysin or pyrotinib monotherapy (Fig. 1h). However, we found no significant difference in the body weight among all treatment groups (Fig. 1i), indicating that neither chrysin nor pyrotinib had any adverse influence on the weight of the mice. These findings collectively elucidated that the combination of pyrotinib plus chrysin yielded a significantly better synergistic therapeutic superiority than chrysin or pyrotinib monotherapy in vitro and in vivo.

### Combination of chrysin plus pyrotinib potentiates autophagy in HER2-positive breast cancer cells by inducing ER stress

**Fig. 2:**
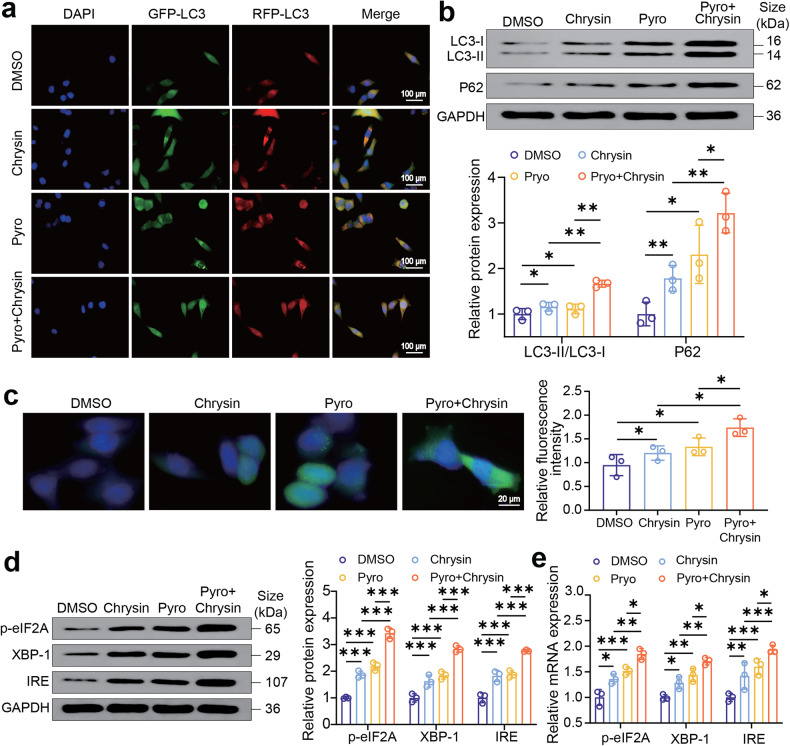
The role of autophagy in the synergistic effect of chrysin and pyrotinib. **a** Autophagy flux of SK-BR-3 cells labeled with mRFP-GFP-LC3 in the different treatment groups. **b** Western blot analysis of the protein expression levels of autophagy markers, LC3-II/LC3-I and P62, in various treatment groups (*n* = 3). **c** Fluorescence imaging detection of ER stress in SK-BR-3 cells after receiving various treatments using ER-Tracker staining (*n* = 3). **d**, **e** Western blot (**d**) and RT-qPCR (**e**) analysis of expression level of ER stress markers, IRE1 or p-eIF2A, in SK-BR-3 cells after various treatments (*n* = 3). All bar values are represented as mean ± SD. *P < 0.05, **P < 0.01, and ***P < 0.001

After confirming a gratifying synergistic effect of pyrotinib and chrysin in anti-HER2-positive breast cancer, we further investigated the underlying mechanism by which pyrotinib in combination with chrysin repressed the survival and proliferation of HER2-positive breast cancer cells. Previous study has shown that autophagy plays a crucial role in lapatinib (a HER2 inhibitor)-induced breast cancer cell death.^24–26^ Therefore, we analyzed if autophagy is highly correlated with the combination treatment-induced cell death. A palpable increase of autophagic flux (fluorescent signals) was detected in SK-BR-3 cells labeled with mRFP-GFP-LC3 in the combination treatment group of pyrotinib and chrysin (Fig. 2a). Besides, we examined the autophagy marker proteins, LC3 and P62 (Fig. 2b), and found that the protein levels of LC3-II/LC3-I and P62 were highest in the pyrotinib + chrysin treatment group, indicating that autophagy is apparently amplified due to the synergistic effect of pyrotinib and chrysin. Chrysin has been demonstrated to trigger autophagy by inducing ER stress in HER2-positive cells.^21^ For this reason, we examined the potential association between the combined drug treatment and ER stress. ER Tracker staining revealed that chrysin or pyrotinib monotherapy led to a weak fluorescence signal intensity, signifying a limited level of ER stress. Conversely, the co-treatment of pyrotinib plus chrysin elicited a notable increase in ER stress within breast tumor cells which was evidenced by intense fluorescence signals (Fig. 2c). In line with this, the combination therapy of pyrotinib plus chrysin significantly potentiated ER stress, as evidenced by apparently increasing ER markers, IRE1, and p-eIF2A in both mRNA and protein levels (Fig. 2d, e). Altogether, these data demonstrated that the combined treatment of pyrotinib plus chrysin could induce ER stress amplification, thereby elevating autophagy within breast cancer cells.

### The G6PD ubiquitination plays a crucial role in the tumorigenesis of HER2-positive breast cancer

**Fig. 3:**
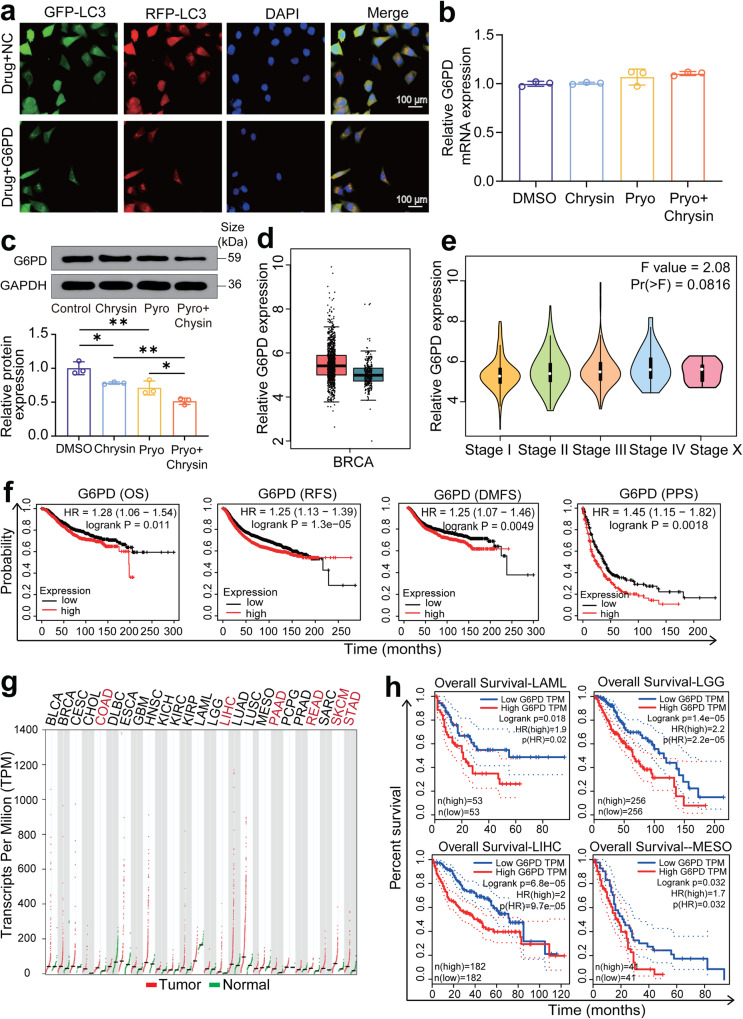
The crucial role of G6PD ubiquitination in the tumorigenesis of HER2-positive breast cancer. **a** Fluorescence images of SK-BR-3 cells subjected to G6PD overexpression for the detection of autophagy. **b**, **c** RT-qPCR (**b**) and western blot (**c**) analysis of relative G6PD mRNA expression in different treatment groups (*n* = 3). **d** The GEPIA database analysis of the G6PD expression level in the TCGA database and in the tumor tissues. **e** The GEPIA database analysis of the G6PD expression in TCGA samples with different tumor stages. **f** TCGA database analysis of the association between G6PD level and the breast cancer prognosis by the Kaplan–Meier plotter. **g** The GEPIA database analysis of the expression level of G6PD in the TCGA database. **h** Correlation analysis between G6PD expression levels and the prognosis of patients with acute myeloid leukemia, brain lower-grade glioma, liver hepatocellular carcinoma, and mesothelioma. All bar values are represented as mean ± SD. *P < 0.05, **P < 0.01

Inhibition of G6PD has been reported to upregulate autophagy in lapatinib-resistant cancer cells.^24^ Next, we examined the potential role of G6PD expression in the pyrotinib and chrysin-enabled breast cancer treatment. We transfected SK-BR-3 cells with the control vector or the vector encoding G6PD, and confirmed a lower level of autophagy in the presence of G6PD overexpression within tumor cells by fluorescence imaging observations (Fig. 3a). RT-qPCR analysis showed that the mRNA expression levels of G6PD did not present an apparent difference between the control and drug therapy group (Fig. 3b); the G6PD protein level slightly decreased in SK-BR-3 cells after solely chrysin or pyrotinib treatment, whereas the lowest level of G6PD protein was detected in the combined therapy group (Fig. 3c). These finding revealed that the combination therapy of pyrotinib plus chrysin does not influence the mRNA expression of G6PD but obviously regulate its protein level. Based on this evidence, we speculate that the combination therapy of pyrotinib and chrysin could be involved in the process of G6PD post-transcriptional modification. In addition, analysis of the GEIPA database with a search of G6PD mRNA identified a similar level of G6PD mRNA between the normal and breast tumor tissues (Fig. 3d, e), which further affirmed our hypothesis. Of special note, TCGA database analysis of the association between G6PD level and breast cancer prognosis demonstrated that high G6PD expression level was closely correlated with poor prognosis of breast cancer (Fig. 3f), which is also verified in a variety of tumors (Fig. 3g, h). With these findings in mind, we hereby confirmed the crucial role of G6PD level in breast cancer prognosis, which inspired us to probe the potential impact of G6PD in the combination therapy of pyrotinib plus chrysin against HER2-positive breast cancer.

### Specific interaction between ZBTB16 and G6PD in the pyrotinib plus chrysin-enabled combination treatment

**Fig. 4:**
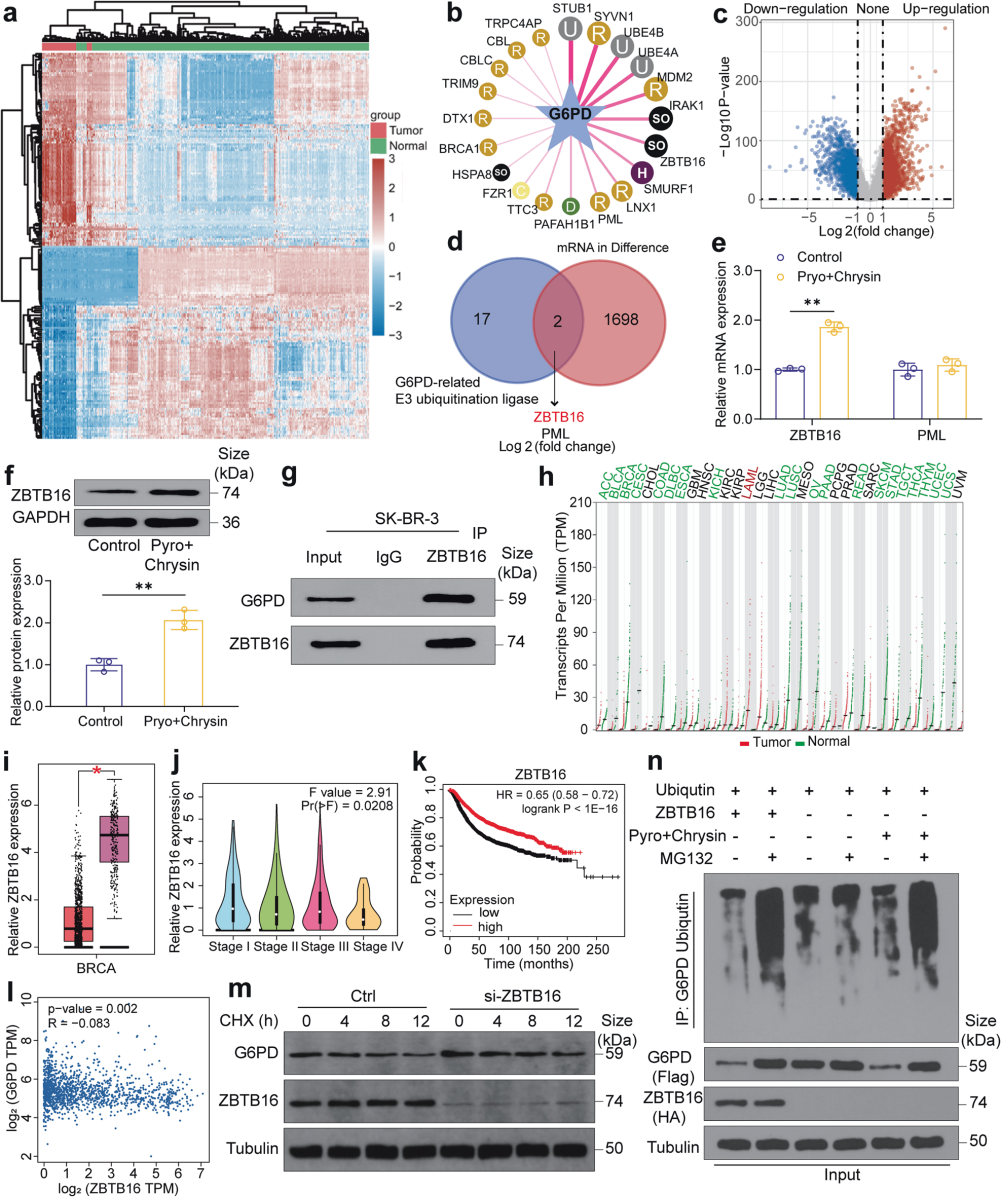
Specific interaction between ZBTB16 and G6PD in the combination treatment of pyrotinib plus chrysin. **a** Heat map analysis of differentially expressed mRNA in breast cancer tissues and adjacent tissues in the TCGA database. **b** Ubibrowser database predicting the E3 ubiquitin ligases that may be involved in the regulation of G6PD ubiquitination. **c** Volcano map analysis of differentially expressed mRNA in breast cancer tissues and adjacent tissues in the TCGA database. **d** Venn diagrams analysis of the intersection genes. **e** RT-qPCR detection of the mRNA expression of PML and ZBTB16 after drug combination treatment (*n* = 3). **f** Western blot detection of the protein expression of ZBTB16 after combined drug treatment (*n* = 3). **g** Interaction analysis between ZBTB16 and G6PD through co-immunoprecipitation experiment. **h**–**j** The GEPIA database determining ZBTB16 expression level in the TCGA database samples with different tumor stages. **k** Relevance of the prognosis of patients with ZBTB16 levels determined by the Kaplan–Meier plotter. **l** The GEPIA database determining the correlation between G6PD and ZBTB16 expression level. **m** Western blot analysis of G6PD protein half-life in SK-BR-3 cells with ZBTB16 silence. Cells were co-incubated with cycloheximide (CHX, 50 μg/ml) for 0, 4, 8, and 12 h. **n** Determination of the ubiquitination of G6PD in cells pretreated with 10 μM MG-132 for 3 h. Cells were transfected with ubiquitin after different treatments. The ubiquitinated G6PD was subjected to immunoprecipitation before western blot with ubiquitin antibody. All bar values are represented as mean ± SD. *P < 0.05, **P < 0.01, and ***P < 0.001

Given that ubiquitination is one of the most important post-translational modifications and ubiquitin binds to target proteins and induces their degradation.^27,28^ Ubibrowser database analysis identified a total of 19 possible E3 ubiquitin ligases that may be involved in the regulation of G6PD ubiquitination (Fig. 4b). TCGA database analysis of differentially expressed mRNAs revealed that the expression levels of 1700 mRNAs were differentially expressed in breast tumor tissues in comparison to their normal counterparts (Fig. 4a, c). The intersection of the two datasets manifested 2 potential targets for HER2-positive breast cancer, zinc finger and BTB/POZ domain-containing family protein 16 (ZBTB16) and promyelocytic leukemia protein (PML), which are known as essential tumor suppressors (Fig. 4d). Afterward, we examined the mRNA expression levels of ZBTB16 and PML in breast cancer tissues. Notably, the combined treatment of pyrotinib and chrysin led to a pronounced increase in the mRNA level of ZBTB16, but not PML, implying that only ZBTB16 was activated by the combination treatment, thus degrading G6PD (Fig. 4e). Western blotting analysis also showed an apparent upregulation of ZBTB16 protein level upon the combined treatment of pyrotinib plus chrysin (Fig. 4f). Additionally, the co-immunoprecipitation confirmed the mutual binding between G6PD and ZBTB16 (Fig. 4g).

Interestingly, the lower level of ZBTB16 was closely correlated with a poorer survival rate (Fig. 4h–l). Thereafter, we analyzed the G6PD protein levels in SK-BR-3 cells after treatment with the control siRNA and siRNA-ZBTB16. It was found that ZBTB16 silence significantly prolonged the half-time of G6PD, suggesting that ZBTB16 promoted the degradation of G6PD (Fig. 4m). As indicated in Fig. 4n, the polyubiquitination of G6PD was enhanced in the presence of ZBTB16 overexpression. Similarly, the combination treatment of pyrotinib and chrysin led to a similar level of polyubiquitination of G6PD. These data indicated the combination treatment of pyrotinib and chrysin could induce ubiquitination, and cause G6PD degradation by targeting and upregulating ZBTB16 level.

### MiR-16-5p is a crucial upregulated miRNA that inversely regulates ZBTB16 in HER2-positive breast cancer, and is highly associated with poor prognosis

**Fig. 5:**
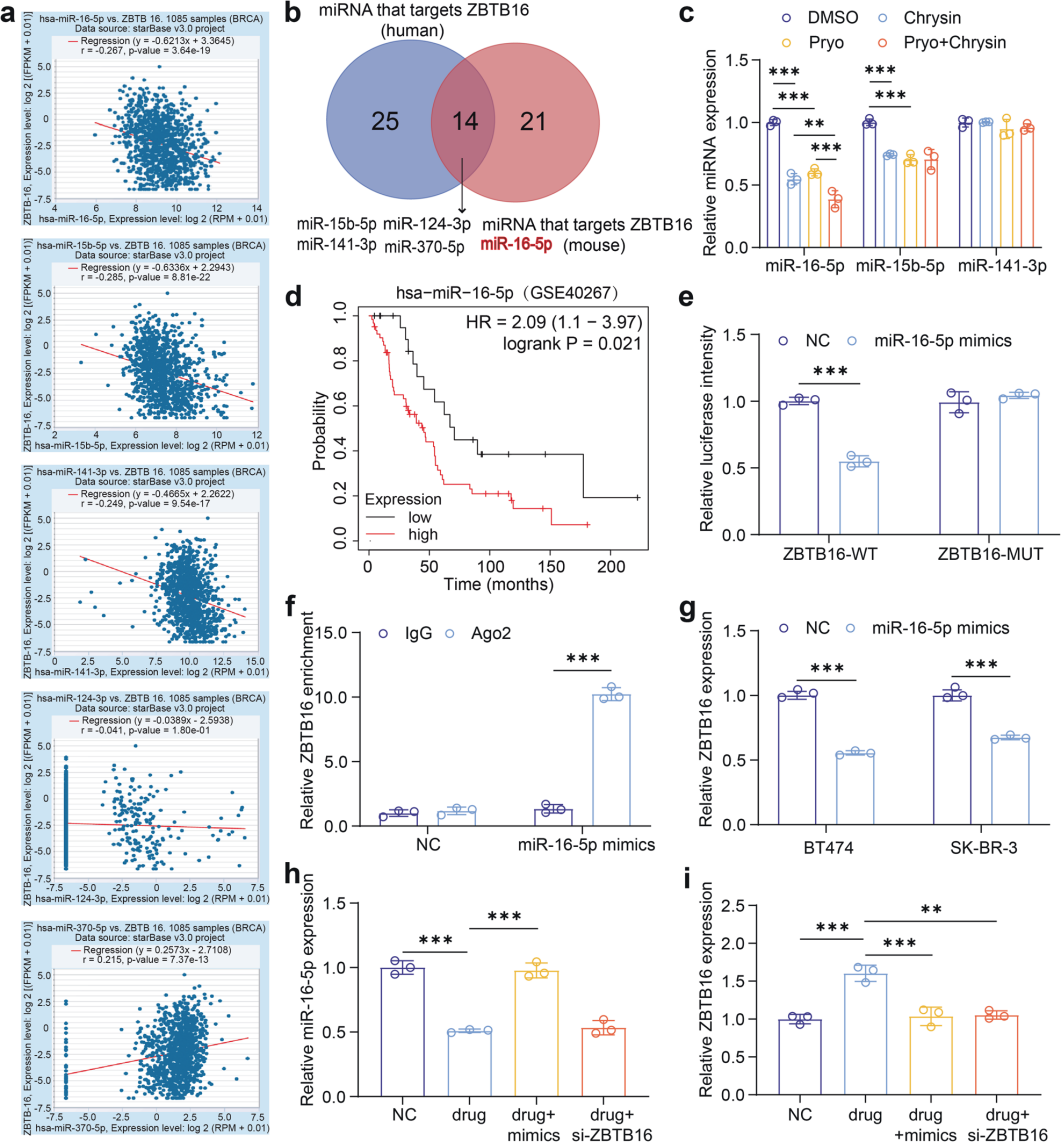
MiR-16-5p is a crucial upregulated miRNA that inversely regulates ZBTB16 in HER2-positive breast cancer. **a** Starbase database analyzing the correlation between the expression of selected microRNAs (miR-16-5p, miR-15b-5p, miR-141-3p, miR-124-3p, and miR-370-5p) and ZBTB16 in breast cancer tissues. **b** Venn diagrams determined the intersection miRNAs that may target ZBTB16. **c** The mRNA levels of miR-15b-5p, miR-141-3p and miR-16-5p after various treatments (*n* = 3). **d** Correlation between miR-16-5p levels and prognosis of breast cancer patients determined by the Kaplan–Meier plotter. **e** Dual-luciferase report verifying the interaction between ZBTB16 and miR-16-5p (*n* = 3). **f** The targeting relation of ZBTB16 and miR-16-5p verified by RIP assay (*n* = 3). **g** The expression of ZBTB16 in BT474 and SK-BR-3 cells after the treatment with NC or miRNA-16-5p mimics (*n* = 3). **h**, **i** The expression of miR-16-5p (**h**) and ZBTB16 (**i**) in SK-BR-3 cells treated NC, combined drug, drug + mimics, and drug + ZBTB16 knockdown (*n* = 3). All bar values are represented as mean ± SD. *P < 0.05, **P < 0.01, and ***P < 0.001

The potential role of microRNA (miRNA) in breast cancer treatment has recently been of growing interest.^29^ The microRNA has been implicated as an early biomarker and potential therapeutic target in oncology research.^30^ For this reason, our study attempted to explore the role of miRNA in the combination treatment of pyrotinib plus chrysin against HER2-positive breast cancer. In response to this idea, we used Targetscan to predict the upstream miRNAs of ZBTB16 and screened them in combination with species conservation. We found that multiple miRNAs (miR-16-5p, miR-15b-5p, miR-141-3p, miR-124-3p, and miR-370-5p) had the highest target binding scores with ZBTB16 (Fig. 5b). Furthermore, we utilized the Starbase database to analyze the correlation between miRNA and ZBTB16 and found that miR-16-5p, miR-15b-5p and miR-141-3p were significantly negatively correlated with ZBTB16 (Fig. 5a). In addition, we found that the level of miR-16-5p and miR-15b-5p decreased with single pyrotinib or chrysin treatment, but only miR-16-5p level further decreased upon the combined treatment of pyrotinib plus chrysin (Fig. 5c). Also, it was revealed that the high expression of miR-16-5p was associated with poor prognosis (Fig. 5d). These results suggested that miR-16-5p may be the target effector molecule that the present study was trying to search for. Finally, dual-luciferase reporter assay validated the possible binding sites for miR-16-5p and ZBTB16 in the Targetscan database (Fig. 5e), where miR-16-5p binds to the 3’UTR region of ZBTB16 and degrades mRNA in the presence of RNA-induced silencing complex (RISC). Similar findings were further supported by RIP experiments (Fig. 5f, g).

**Fig. 6:**
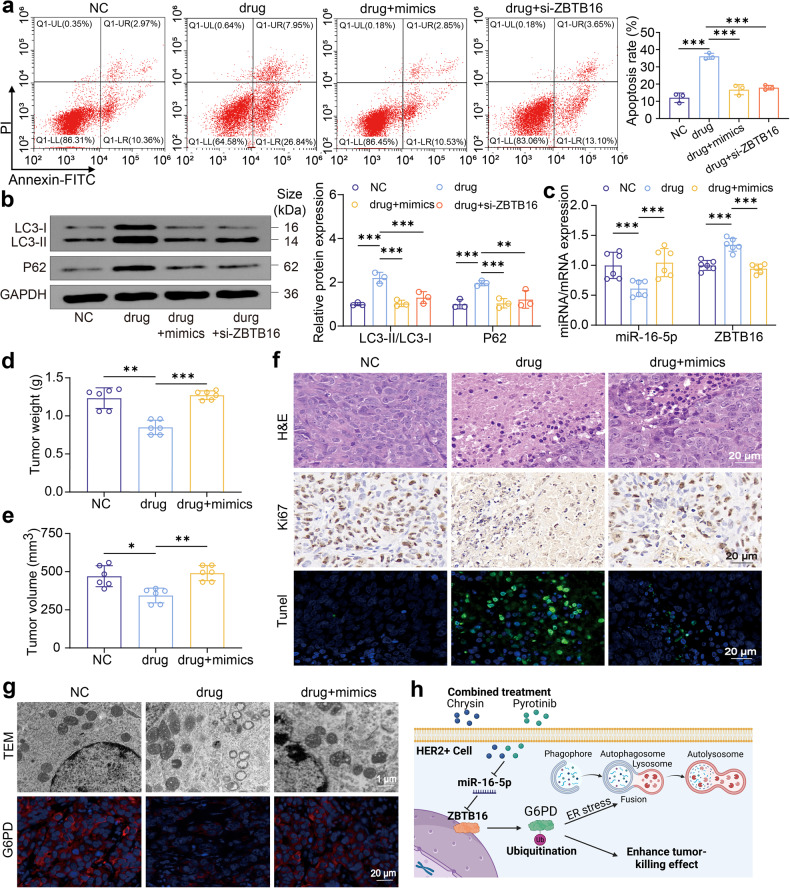
The impact of miR-16-5p overexpression on the therapeutic efficacy of the pyrotinib + chrysin treatment against HER2-positive breast cancer. **a** Cell apoptosis detection of SK-BR-3 cells after different treatments by flow cytometry (*n* = 3). **b** Western blot detection of the expression levels of autophagy markers (LC3B and P62) in different treatment groups (*n* = 3). **c**–**e** The expression levels of miR-16-5p and ZBTB16 in tumor tissues (**c**), tumor weight (**d**), and tumor volume (**e**) from subcutaneous SK-BR-3 tumor xenograft models after three different treatments (*n* = 6). **f** H&E, Ki67, and Tunnel immunohistochemical staining of tumor sections from the treated mice in various groups determining tumor proliferation and apoptosis. **g** Representative TEM images (upper panel) of autophagosomes of tumors in various treatment groups (×20000). Immunohistochemical staining images (lower panel) of tumor slices for the determination of G6PD level. **h** Schematic illustration of the combined treatment of pyrotinib and chrysin synergistically potentiating autophagy in HER2-positive breast cancer cells via regulating miR-16-5p/ZBTB16/G6PD axis. All bar values are represented as mean ± SD. *P < 0.05, **P < 0.01, and ***P < 0.001

To elucidate the interaction of miR-16-5p and ZBTB16 in the combined treatment of pyrotinib plus chrysin, we constructed SK-BR-3 cell models with miR-16-5p overexpression or ZBTB16 knockdown. The results showed that the pyrotinib + chrysin group led to a palpable downregulation of miR-16-5p, which was counteracted by the addition of mimics (Fig. 5h). Interestingly, the pyrotinib + chrysin treatment resulted in a notable upregulation of ZBTB16 expression, whereas ZBTB16 knockdown via siRNA-ZBTB16 could neutralize the effect (Fig. 5i). In line with this, the miR-16-5p overexpression or ZBTB16 knockdown correspondingly decreased the apoptosis rate induced by the combined treatment (Fig. 6a). It was noted that the pyrotinib + chrysin treatment potentiated autophagy in HER2-positive breast cancer cells, whereas the overexpression of miR-16-5p or knockdown of ZBTB16 apparently decreased autophagy, as indicated by simultaneously decreased ratio of LC3-II/LC3-I (Fig. 6b).

To further validate the role of miR-16-5p overexpression on the therapeutic efficacy of the pyrotinib + chrysin treatment, the xenograft mice models were established. It was found that the combined therapy with pyrotinib and chrysin apparently increased ZBTB16 level in tumor tissues (Fig. 6c); this effect could be reversed by miR-16-5p overexpression via its mimics, which is in high accordance with the results of in vitro assays. Notably, the tumor volume and tumor weight in mice treated with pyrotinib + chrysin and miR-16-5p mimics were significantly increased compared with those without miR-16-5p treatment (Fig. 6d, e). As indicated by H&E, Ki67 and Tunnel staining, the combined drug (pyrotinib + chrysin) plus miR-16-5p mimics resulted in an apparent increase in tumor cells (Fig. 6f). Furthermore, the combined therapy group presented a large number of autophagic vacuoles, whereas only a few autophagic vacuoles were observed in the combined medications plus miR-16-5p overexpression treatment group (Fig. 6g). The lower level of G6PD signals was also observed in the pyrotinib + chrysin plus miR-16-5p mimics treatment group (Fig. 6g). In agreement with observations, it is clearly demonstrated that the combination treatment of pyrotinib and chrysin synergistically potentiates autophagy in HER2-positive breast cancer cells via regulating miR-16-5p/ZBTB16/G6PD axis (Fig. 6h). As a result, these data suggested that miR-16-5p overexpression significantly weakened the overall therapeutic efficacy of pyrotinib plus chrysin, which provide new insights for treating breast cancer against miR-16-5p.

## Discussion

The amplification or overexpression of HER2 occurs in roughly 20% of all breast cancer cases.^31^ The overexpression of this transmembrane tyrosine kinase receptors is considered to promote abnormal growth and invasion of breast cancer cells via triggering dimer formation bias of the ErbB family of receptors and aberrantly activating downstream pathways, leading to aggressive tumor cells, focal progression and poor prognosis.^5^ With a deeper insight into the molecular mechanisms of HER2-positive breast cancer, a range of HER2-targeted drugs have been well developed.^32–34^ Strikingly, pyrotinib has been demonstrated to dramatically improve the poor prognosis of patients with HER2-positive breast cancer, opening a new era of targeted therapy.^35^ Nevertheless, the effectiveness of pyrotinib monotherapy remains restricted due to tumor heterogeneity and primary or acquired drug resistance, which inevitably limits its clinical efficiency. In this study, we determined that pyrotinib combined with chrysin could elicit a synergistic superiority in inhibiting HER2-positive breast cancer in vitro and in vitro than single pyrotinib or chrysin treatment via the regulation of miR-16-5p/ZBTB16/G6PD axis.

Intensified levels of autophagy may mechanistically be responsible for enhanced inhibitory efficacy of the combination with pyrotinib and chrysin in the treatment of HER2-positive breast cancer. Autophagy is a common spontaneous metabolic activity in cell homeostasis, in which cytoplasmic contents or other organelles are engulfed by bilayer membrane structure, and then degraded by the fusion of autophagosome and lysosome.^36^ Autophagy deficiency may play an essential role in the development and progression of HER2-positive breast cancer, which is also associated with resistance to targeted HER2 therapy.^37^ Recent reports revealed that one-third of breast cancer patients with autophagy gene beclin 1/BECN1 had monoallelic deletions, resulting in significantly reduced levels of autophagy.^38^ BECN1 allele deletion causes a rise in breast cancers in mice, and its enhanced expression markedly inhibits the progression of human breast cancer xenografts. Another in vitro study conducted by Chen et al. proved that autophagy is a potential novel therapeutic target for reversing lapatinib resistance of HER2-positive breast cancer cells.^39^ Our previous studies have demonstrated that chrysin is capable of potentiating autophagy in tumor cells by activating ER stress.^21^ In the present study, we uncovered that the combination treatment of pyrotinib and chrysin could yield a synergistic effect in apparently increasing autophagy within HER2-positive tumor cells by inducing ER stress.

Inhibition of G6PD has been proven to trigger the upregulation of autophagy in lapatinib-resistant cancer cells.^24^ Consistent with this finding, our study also demonstrated that the combined treatment of pyrotinib and chrysin induced an apparently lower level of autophagy within HER2-positive breast cancer cells in the presence of G6PD overexpression. In the meanwhile, we observed for the first time that the combination treatment of pyrotinib and chrysin does not influence the G6PD mRNA expression but obviously regulates the G6PD protein level. It is therefore reasonable to speculate that the drug combination therapy may be involved in the process of G6PD post-transcriptional modification. Besides, TCGA database analysis revealed that high G6PD expression level was closely correlated with poor prognosis of breast cancer, which is also verified in a variety of tumors. Afterward, we exploited bioinformatic analysis by ubibrowser and TCGA database to search for potential targets for HER2-positive breast cancer. Further investigations validated zinc finger and BTB/POZ domain-containing family protein 16 (ZBTB16) as an E3 ubiquitination ligase that may be involved in the regulation of G6PD ubiquitination. Previous studies suggested that ZBTB16 suppresses breast cancer development and progression by upregulating ZBTB28 and inhibiting ZBTB27.^40^ However, the specific interactions between ZBTB16 and G6PD, as well as the role of ZBTB16 in G6PD ubiquitination remain unclear. Here, our findings determined that the combination treatment of pyrotinib and chrysin induced G6PD ubiquitination, thereby causing G6PD degradation by targeting and upregulating ZBTB16.

It is generally established that microRNA (miRNA), an RNA cluster with 18–25 nucleotides, has a biological function by incompletely binding to the target gene to affect the expression of target genes.^29,30^ A number of studies confirmed that numerous miRNAs play a crucial role in tumor progression and development.^30,41^ Previous studies indicated that miR-16-5p may be involved in regulating the expression of AKT and suppressing breast cancer progression by decreasing AKT3 to restrain the NF-κB pathway.^42,43^ In this study, we predicted and screened the upstream miRNAs of ZBTB16 through Targetscan, and verified a pivotal function of miR-16-5p as an effector molecule in HER2-positive breast cancer. In addition to that, we identified that miR-16-5p is a potential upstream regulatory target of ZBTB16, and it showed a significant inverse correlation with ZBTB16. We found that the combination treatment of pyrotinib and chrysin dampened the G6PD expression in HER2-positive breast cancer cells, whereas overexpression of miR-16-5p or knockdown of ZBTB16 increased the G6PD expression in breast cancer cells, along with apparently decreased autophagy. In line with that, an identical therapeutic trend was also verified in mice xenograft models. Together, these findings evidence that miR-16-5p overexpression promotes HER2-positive tumorigenesis, and apparently impairs the efficacy of the combination therapy of pyrotinib and chrysin, which provides new insights into targeting miR-16-5p for the treatment of HER2-positive breast cancer.

Currently, the generally accepted treatment strategy is combining anti-HER2 therapy with other targeted drugs to potentiate therapeutic efficacy via overcoming drug resistance but decreasing drug-induced toxicity. However, our present study still presents several restrictions. First, we only focused on the role of the miR-16-5p/ZBTB16/G6PD axis in regulating the anticancer efficacy of combined treatment of pyrotinib and chrysin, but other possible pathways involved in this process were not elucidated. Second, we do not identify the individual contributions of each drug to autophagy regulation and how they may interact with each other to synergistically enhance their overall anticancer effect.

In summary, our study identifies for the first time the tremendous therapeutic potential of pyrotinib combined with chrysin in treating HER2-positive breast cancer but also sheds light on the functional role of miR-16-5p/ZBTB16/G6PD axis in the anti-HER2 therapeutic process. Together, our study presents a novel treatment option, pyrotinib combined with chrysin, in treating HER2-positive breast cancer.

## Materials and methods

### Cell lines

Human breast cancer cell lines BT474, MDA-MB-231, SK-BR-3, and MCF-7 were obtained from BeiNa biological company (China). BT474 and SK-BR-3 cells were cultivated in 10% FBS-containing DMEM medium in a humidified atmosphere with 5% CO_2_ at 37 °C. Meanwhile, a 10% FBS-supplemented RPMI1640 medium was used for the maintenance of MDA-MB-231 and MCF-7 cells.

### Cell proliferation assay

For the CCK-8 assay, the relative viability of cells after various treatments was measured using the Cell Counting Kit (Yeasen, China). Finally, the absorbance value (optical density) of each well was measured by a microplate reader at the wavelength of 450 nm.

### Flow cytometry

SK-BR-3 cells (1 × 10^6^) were inoculated into 6-well plates and treated with PBS, combined drug treatment (chrysin (20 μM) + pyrotinib (10 nM)), drug + mimics treatment, and drug + si-ZBTB16 treatment, respectively. At 48 h post-treatment, the cells were collected and subjected to multiple centrifugations to completely remove the supernatant.

Apoptosis assays were performed on SK-BR-3 cells by resuspending them in a binding buffer and double-staining them with Annexin V and propidium iodide (PI) (BestBio, China). After that, the cell apoptosis level was identified through a flow cytometer (Beckman Coulter, USA).

To perform cell cycle analysis, SK-BR-3 cells after various treatments were digested with 0.25% trypsin without EDTA. After the digestion was terminated, the supernatant was discarded by centrifugations at 1500 rpm for 5 min; the cells were collected and then rinsed twice with PBS before resuspending them in 100 μl of PBS. Then, 700 μl of pre-cooled 80% ethanol was slowly injected into the cell samples to make a final concentration of 70%. The cells were then fixed at 4 °C for at least 4 h, followed by multiple centrifugations at 1500 rpm for 5 min and twice rinses with pre-cooled PBS. After incubation at 37 °C in 10 μl of RNase solution (1 mg/ml) for 30 min, the cells were stained with PI (250 μg/ml) at 4 °C for 45 min in darkness. Finally, the cell cycle was detected by flow cytometry.

### Autophagy flux detection

After various treatments, SK-BR-3 cells were transfected with mRFP-GFP-LC3 (Wanlei bio, China) and stained with DAPI solution for 5 min at room temperature before mounting the cover slides. Each glass slide from various treatment groups was photographed using a confocal laser microscope.

### Bioinformatics analysis

The ubibrowser database (http://ubibrowser.ncpsb.org.cn/ubibrowser/) was utilized to predict the E3 ubiquitin ligases. Similarly, for the analysis of the prognosis of the target molecule, a Kaplan–Meier plotter was applied (https://kmplot.com/analysis/).

### The cancer genome atlas (TCGA) database analysis

R software was used to study the differential mRNA expression of TCGA in this study. The criteria of adjusted *P* < 0.05 and log2 (fold change)> 1 or log2 (fold change) <−1 were applied to screen for differential expression of mRNA.

### Dual-luciferase reporter assay

The Targetscan database (http://www.targetscan.org/vert_72/) was used to predict the targeted binding site of miR-16-5p on ZBTB16. The predicted miR-16-5p binding site within wild-type or mutant ZBTB16 3’UTR was amplified via PCR and then inserted into the pMIR-reporter plasmid. Two luciferase reporter plasmids that contained ZBTB16 were transfected into cells, alongside the simultaneous transfection of miR-16-5p mimics. Following incubation for 48 h, we determined the luminescence through a dual luciferase detection kit (Promega, USA) in accordance with the manufacturer’s instructions.

### G6PD deubiquitination assay

Ubiquitinated G6PD (Ub-G6PD) was incubated with recombinant Flag-ZBTB16 in 30 μl of deubiquitination buffer containing Tris-HCl (50 mM, PH 7.4), NaCl (150 mM), DTT (10 mM), and MgCl_2_ (5 mM) at 37 °C for 2 h. Thereafter, the reaction was terminated by adding 25 μl of 2×SDS sample buffer and boiling it for 5 min. G6PD was ultimately immunoprecipitated with an anti-HA antibody and examined by western blotting.

### Cell transfection

G6PD overexpression plasmid and miR-16-5p mimic were designed and synthesized by GenePharma (Shanghai, China). miR-NC inhibitor: 5ʹ-UAGCAGCACGUAAAUAUUGGCG-3ʹ; miR-NC mimic: 5ʹ-GGAACUUAGCCACUGUGAAUU-3ʹ; miR-16-5p mimic: 5ʹ-UUCUCCGAACGUGUCACGUTT-3ʹ. Lentiviral short hairpin RNA targeting ZBTB16 (si-ZBTB16) and its negative control, were produced by Genechem (Shanghai, China). Cells were transfected with designated plasmids by employing Lipofectamine 2000 (Invitrogen, USA).

### Real-time PCR (RT-qPCR)

Total RNA was extracted and collected by TRIzol (Ambion, USA), and complementary DNA (cDNA) was generated with an RNA-to-cDNA kit (Thermo Fisher Scientific, MA, USA) using the total RNA. Then, the expression levels of transcripts were determined with SYBR Green qPCR Master Mix (MedChem Express, NJ, USA) on a 900HT Fast Real-Time PCR Detection System (Thermo Fisher Scientific, MA, USA). The relative gene expression was quantified and normalized to GAPDH or U6 expression by the 2^−^^ΔΔCt^ method. Primer sequences are illustrated in Supplementary Table 1.

### Western blot (WB)

Cells were lysed and the protein was extracted by the addition of RIPA buffer (Beyotime, China). Then, the cell lysate was subjected to sodium dodecyl sulfate-polyacrylamide gel electrophoresis (SDS-PAGE) gel prior to transfer to a polyvinylidene fluoride (PVDF) membrane (Millipore). Subsequently, the membrane was incubated with blocking buffer-diluted primary antibodies, including p-HER2 (ab201013), HER2 (ab237715), LC3 (ab192890), P62 (ab109012), p-eIEFA (ab157478), XBP-1 (ab37152), IRE (ab37073), G6PD (ab210702), ZBTB16 (ab104854) and GAPDH (ab8245), at 4 °C overnight. After washing in Tris-buffered saline with 0.05% Tween 20 (TBST), hybridized membranes with horseradish peroxidase (HRP)-linked antibody goat anti-rabbit IgG (1:2000, Abcam, UK) were incubated for another 1 h. Finally, the enhanced chemiluminescence reagent was used to visualize the immunoreactive bands.

### Co-immunoprecipitation (Co-IP)

In short, SK-BR-3 cells were transfected with ZBTB16 plasmid, and the total protein was extracted using ice-cold low-salt lysis buffer, followed by rotation with anti-G6PD or IgG antibody with a constant speed (60 r/min) at 4 °C overnight. Then, the Co-IP complexes were precipitated with protein A/G magnetic beads (Merck, Germany), and the magnetic beads were rinsed thrice using a low-salt lysis buffer. To this end, co-immunoprecipitation complexes were determined by western blotting.

### Ribonucleoprotein immunoprecipitation (RIP) assay

To put it briefly, the cell lysate was blocked using Protein G magnetic beads and treated with anti-AGO G magnetic beads (Thermo Fisher Scientific, USA) at 4 °C for 1.5 h. After centrifugation at 700 × *g* for 60 s, the beads were rinsed 6 times with RIPA buffer and resuspended in 50 mmol/l Tris-HCl at pH 7.0. Following magnetic beads crosslinking at 70 °C for 45 min, RNA Co-IP was performed with anti-AGO antibody, and RT-qPCR was carried out to quantify the target molecule.

### Animal model

The animal study conformed to the ethical and scientific guidelines approved by the Animal Ethics Committee of Sichuan University (Permit Number: 20230222058). Seventy BALB/c nude mice were purchased from Charles River Laboratories (Shanghai, China). In this study, subcutaneous injections of BT-474 or SK-BR-3 cells in a total of 2 × 10^6^ were given into the lower abdomens of nude mice. Following the injection, the tumor volume was measured every 3 days, and the volume was calculated as per this formula: volume = (width^2^ × length)/2. Once the tumor volume reached the prescribed size (100 mm^3^), these nude mice were gavaged with normal saline, chrysin (50 mg/kg), pyrotinib (10 mg/kg), or their combination once a day during the 21-day treatment period. Meanwhile, to analyze the effect of miR-16-5p expression level on antitumor efficacy, some SK-BR-3 tumor-carrying nude mice were further divided into NC group, drug group (chrysin (50 mg/kg) plus pyrotinib (10 mg/kg)) and drug + mimics group (miR-16-5p mimic was injected into the tumor tissue after same drug treatment every 3 days). In the end, the mice were sacrificed, and the tumor tissues were harvested and stored in liquid nitrogen.

### Ki‑67 and TUNEL staining

For each treatment group, the tumor tissues of mice were paraffin-embedded and sliced, followed by staining with a Ki-67 immunohistochemistry kit or a Tunnel analysis kit as per the manufacturer’s instructions.

### Hematoxylin and eosin (H&E) staining

For each treatment group, the tumor tissues of mice were harvested and embedded in paraffin after deparaffinization and dehydration. Thereafter, the specimens were sliced and stained with hematoxylin and eosin and finally imaged with a microscope to assess cell morphology and tissue architecture.

### Statistical analysis

All data statistics were performed using SPSS22.0 and GraphPad Prism 9 software. The experimental data were presented as mean ± standard deviation (SD). The comparison between two groups is analyzed by Student’s *t*-test, and the comparison between multiple groups is compared by one-way analysis of variance (ANOVA) followed by Tukey’s multiple comparisons test (where applicable). The levels of significant difference among the groups were denoted by *P < 0.05, **P < 0.01, and ***P < 0.001.

### Supplementary information

Supplementary Materials

## Data Availability

The original data presented in the study are included in the article, further inquiries can be directed to the corresponding author.
