# Supplementary material for: Pyrotinib and chrysin synergistically potentiate autophagy in HER2-positive breast cancer
Source: Signal Transduct Target Ther. 2023 Dec 18;8:463. doi: 10.1038/s41392-023-01689-w (PMC10728098; doi:10.1038/s41392-023-01689-w)
Supplement: Supplementary file 1 — Supplementary Materials [file 41392_2023_1689_MOESM1_ESM.docx]

Supplementary Materials for

Pyrotinib and Chrysin Synergistically Potentiate Autophagy in HER2-Positive Breast Cancer

Xiaoxiao Liu^1,2,†^, Xing Zhang^3,†^, Zhiying Shao^4,†^, Xiaorong Zhong^1,†^, Xin Ding^2^, Liang Wu^5^, Jie Chen^6^, Ping He^1^, Yan Cheng^1^, Kunrui Zhu^1^, Dan Zheng^1^, Jing Jing^7,*^, Ting Luo^1,*^

*Correspondence: Jing [Jing (jingjing@wchscu.cn),](mailto:Jing(jingjing@wchscu.cn),) Ting Luo (luoting[@wchscu.cn](mailto:@wchscu.cn))

^†^These authors contributed equally: Xiaoxiao Liu, Xing Zhang, Zhiying Shao and Xiaorong Zhong

**This PDF file includes:**

Supplementary Table 1

**Supplementary Table 1. Primer sequence**

|  | **Primer Sequence** |
| --- | --- |
| miR-16-5p forward | 5'-TAGCAGCACGTAAATATTGGCG-3 ' |
| miR-16-5p reverse | 5'-CTCAACTGGTGTCGTGGA-3' |
| U6 forward | 5’-CTCGCTTCGGCAGCACATA-3’ |
| U6 reverse | 5’-AACGATTCACGAATTTGCGT-3’ |
| G6PDforward | 5’-AAGAACGTGAAGCTCCCTGA-3’ |
| G6PDreverse | 5’-AATATAGGGGATGGGCTTGG-3’ |
| HER2 forward | 5’-CAGAAGATCCGGAAGTACAC -3’ |
| HER2 reverse | 5’-ATACACCAGTTCAGCAGGTC -3’ |
| P62 forward | 5’-TGTGGAACATGGAGGGAAG-3’ |
| P62 reverse | 5’-TGTGCCTGTGCTGGAACTTTC-3’ |
| LC3 forward | 5’-TTGGTCAAGATCATCCGGC-3’ |
| LC3 reverse | 5’-GCTCACCATGCTGTGCTGG-3’ |
| ZBTB16forward | 5’-TTTCAGCCATGAGTCCCACC-3’ |
| ZBTB16reverse | 5’-CTCAACCTTGTCCCCCATCC-3’ |
| GAPDH forward | 5’-GAAGGTGAAGGTCGGAGTC-3’ |
| GAPDH reverse | 5’-GAAGATGGTGATGGGATTTC-3’ |
